# Risk factors for the occurrence and progression of diabetic retinopathy in children and adolescents: a systematic review and meta-analysis

**DOI:** 10.3389/fpubh.2026.1832691

**Published:** 2026-05-21

**Authors:** Jiaojiao Jiang, Wanqing Guo, Zhixiang Ding

**Affiliations:** Department of Ophthalmology, The First Affiliated Hospital of Guilin Medical University, Guilin, China

**Keywords:** children and adolescents, diabetic retinopathy, meta-analysis, risk factors, systematic review

## Abstract

**Background:**

Diabetic retinopathy (DR) is a severe microvascular complication in children and adolescents with diabetes, causing irreversible visual impairment. Despite increasing pediatric diabetes prevalence, evidence on DR risk factors in this population remains limited. This study aimed to systematically evaluate risk factors for DR onset and progression to support early screening and stratified management.

**Methods:**

Systematic review and meta-analysis was registered with PROSPERO (CRD420261330671). Databases including PubMed, Web of Science, Embase, and Scopus were searched from inception to March 2026. Eligible studies were cohort studies involving patients aged ≤ 20 years. Study quality was assessed using the Newcastle-Ottawa Scale. Analyses were performed in R 4.2.3 with random-effects model; meta-regression, subgroup analyses, and trim-and-fill method were used to explore heterogeneity and publication bias.

**Results:**

Twenty-eight cohort studies with 71,697 participants (93.84% T1DM) were included. Meta-analysis demonstrated that elevated glycated hemoglobin (HbA1c) was the strongest predictor of DR onset and progression. In the random-effects model, each 1% increase in HbA1c was associated with a 78% higher risk of DR onset (HR = 1.76, 95% CI: 1.45–2.14) and a 65% higher risk of DR progression (HR = 1.65, 95% CI: 1.15–2.37). Elevated systolic blood pressure (HR = 1.17), longer diabetes duration (HR = 1.38), and younger age at diabetes onset (HR = 0.84) were associated with DR onset, although these associations did not reach statistical significance in random-effects models. Meta-regression revealed that exposure metric and sample size were the main sources of heterogeneity, with small-sample studies systematically overestimating effect sizes. The trim-and-fill method indicated significant publication bias for the association between HbA1c and DR, and the pooled effect became statistically nonsignificant after bias correction.

**Conclusions:**

HbA1c is a key associated factor for pediatric DR, but its estimated effect is significantly affected by publication bias and high heterogeneity, and the pooled effect became non-significant after bias correction. Therefore, conclusions should be drawn cautiously.

**Systematic review registration:**

https://www.crd.york.ac.uk/PROSPERO/view/CRD420261330671, identifier: CRD420261330671.

## 1 Introduction

The global diabetes epidemic has grown increasingly severe, representing one of the fastest-growing major public health challenges of the 21st century. According to the latest data from the 11th edition of the International Diabetes Federation (IDF) Diabetes Atlas, the global prevalence of diabetes among adults aged 20–79 years reached 11.11% in 2024. The total number of cases rose to 589 million, and it is projected to increase to 853 million by 2050 (1). Of great concern, the burden of diabetes is no longer confined to adults. Globally, approximately 9.5 million people were living with type 1 diabetes (T1DM) in 2024. Of these, 1.85 million were children and adolescents aged 0–19 years (2). A more alarming trend is the rising incidence of type 2 diabetes (T2DM) among young people. It has increased at an annual rate of 3.01%. Between 1990 and 2021, the incidence of T2DM among those aged 10–24 years rose from 56.02 to 123.86 per 100,000 population. This downward shift in the age of diabetes onset exposes younger generations to a longer disease course and a longer period of complication risk (3).

Diabetic retinopathy (DR) is one of the most damaging chronic microvascular complications of diabetes. Its pathological basis involves retinal microvascular damage and neural structural injury caused by chronic hyperglycemia (4). According to global epidemiological data, the overall prevalence of DR among people with diabetes was 22.27% in 2020. Vision-threatening diabetic retinopathy (VTDR) accounted for 6.17%, and clinically significant macular edema (CSME) accounted for 4.07%. Based on current trends, the global number of people with DR is projected to reach 160 million by 2045. Among these, cases of VTDR and CSME will rise to 44.82 million and 28.61 million, respectively (5). DR is not only a major global public health issue. It is also the leading cause of irreversible blindness among people aged 20–74 years in the working-age population (6). Compared with adult patients, children and adolescents with diabetes have a longer disease course and earlier exposure to hyperglycemia. They face a significantly higher risk of DR onset and faster disease progression. Once DR advances to severe stages, it can lead to permanent visual disability. It also seriously impairs children's growth and development, academic performance, daily life, and long-term quality of life (7).

In recent years, several reviews have only provided qualitative summaries of risk factors for DR in children and adolescents. Glycated hemoglobin (HbA1c), diabetes duration, abnormal blood pressure, and dyslipidemia have been preliminarily identified as core associated factors (8, 9). However, these reviews did not perform quantitative meta-analyses. Moreover, the original cohort studies included in these reviews show substantial inconsistencies. They differ greatly in sample size, follow-up period, study design, and definitions of risk factors. As a result, no unified consensus has been reached on the effect sizes and clinical warning thresholds of key indicators. In addition, some relevant studies adopt a cross-sectional design. This design cannot accurately clarify the causal temporal relationship for the onset and progression of DR. Some studies focus only on a single risk factor, lacking a comprehensive assessment of the combined effects of multiple factors. These limitations make it difficult for clinicians and public health policymakers to obtain high-quality, robust evidence. They also prevent the development of targeted strategies for early screening and stratified management of DR in children and adolescents. Currently, most systematic reviews and meta-analyses of DR risk factors focus on adult populations. A small number of meta-analyses have specifically investigated DR risk factors among pregnant women. However, high-quality quantitative synthesis targeting children and adolescents remains lacking (10).

Therefore, this study aims to systematically search for relevant domestic and international cohort studies. We will systematically evaluate the risk factors for DR onset and progression in children and adolescents with diabetes. We will further conduct a meta-analysis to explore the risk trends of key indicators. This study intends to provide high-quality evidence for the long-term management of diabetes, complication prevention, and vision protection in pediatric patients.

## 2 Materials and methods

### 2.1 Study design and registration

This systematic review and meta-analysis has been registered with the international prospective register of systematic reviews PROSPERO (registration number: CRD420261330671). The entire study was conducted in strict accordance with the Preferred Reporting Items for Systematic Reviews and Meta-Analyses (PRISMA 2020) guideline (11). This approach ensures the transparency, reproducibility, and rigor of the study.

### 2.2 Literature search strategy

Computerized searches were performed in international English databases to comprehensively collect relevant literature. The search timeframe was from the inception of each database to March 2, 2026. The English databases included PubMed, Web of Science, Embase, and Scopus. A combined search strategy of subject headings and free-text words was adopted. Search terms were standardized with the Medical Subject Headings (MeSH) to ensure search accuracy. English search terms included: “children” “adolescent” “youth” “pediatric” “diabetic retinopathy” “retinopathy diabetic” “diabetes mellitus” “diabetes” “risk factors” “predictors” “incidence”, and “progression”. Boolean operators “AND” and “OR” were used for search combinations.

Taking the PubMed database as an example, the search strategy was as follows:((“diabetic retinopathy”[MeSH Terms] OR “diabetic retinopathy”[Title/Abstract] OR “retinopathy, diabetic”[Title/Abstract]) AND (“child”[MeSH Terms] OR “adolescent”[MeSH Terms] OR “young adult”[MeSH Terms] OR “pediatric”[Title/Abstract] OR “pediatric”[Title/Abstract] OR “childhood”[Title/Abstract] OR “adolescence”[Title/Abstract] OR “youth”[Title/Abstract]) AND (“diabetes mellitus”[MeSH Terms] OR “diabetes”[Title/Abstract] OR “juvenile diabetes”[Title/Abstract] OR “childhood-onset diabetes”[Title/Abstract])) AND (“risk factor”[Title/Abstract] OR “predictor”[Title/Abstract] OR “incidence”[Title/Abstract] OR “progression”[Title/Abstract]).

In addition, the detailed search strategies of all databases are presented in Supplementary File S1.

### 2.3 Study inclusion and exclusion criteria

This study strictly followed the PICO principle to develop literature inclusion and exclusion criteria. We aimed to accurately screen original studies that matched the research purpose and ensure homogeneity and relevance of included studies. Specific criteria are listed below.

Inclusion Criteria: We included internationally published observational studies, including prospective cohort, retrospective cohort, and case-control studies. ① Study participants were diabetes patients aged ≤ 20 years (including type 1 and type 2 diabetes), diagnosed using internationally recognized standards such as American Diabetes Association (ADA) criteria. ② Exposure factors included all potential risk factors linked toDR onset and progression, such as diabetes duration, glycemic control, blood pressure, blood lipids, body mass index (BMI), age at diabetes onset, sex, and diabetes type. Control groups were set per standard observational study protocols. ③ Outcome measures were clearly defined, with DR onset as the primary outcome and DR progression as the secondary outcome. Outcomes were graded using internationally accepted scales, and complete data were available to extract effect sizes (odds ratio [OR], relative risk [RR], hazard ratio [HR] and 95% confidence interval [CI]).

Exclusion Criteria: We excluded ineligible studies as follows: ① basic experimental studies (animal studies, *in vitro* cell assays, gene-level basic research); ② review articles and non-original literature (systematic reviews, meta-analyses, narrative reviews, conference abstracts, editorials, letters, dissertations); ③ cross-sectional studies, which cannot establish the temporal relationship between risk factors and DR onset/progression; ④ studies involving participants over 20 years old without separate data for those ≤ 20 years old; ⑤ patients with severe ocular diseases (congenital cataract, glaucoma, retinal detachment), severe systemic disorders (severe hepatic/renal insufficiency, autoimmune diseases, malignant tumors), or pregnant/lactating individuals. Congenital cataract was excluded because it impairs adequate visualization of the retina, preventing reliable assessment of DR status. ⑥ studies with a total sample size < 50 due to concerns about low statistical power; ⑦ studies reporting only unadjusted effect estimates without controlling for key confounding factors; ⑧ studies with unclear or non-standard DR diagnosis criteria; ⑨ studies with incomplete data that could not provide valid effect sizes (and no author-supplemented data); ⑩ low-quality studies per standardized quality assessment; ⑪ non-English publications; ⑫ studies unable to separate DR outcomes from other eye conditions; ⑬ duplicate or overlapping publications.

### 2.4 Literature screening and data extraction

Two systematically trained researchers, Jiaojiao Jiang and Wanqing Guo, independently completed literature screening in strict accordance with the inclusion and exclusion criteria across three stages: first, preliminary screening, in which the researchers browsed the titles and abstracts and excluded obviously ineligible studies such as animal experiments, review articles, and studies with inappropriate study populations; second, secondary screening, in which full texts of studies passing preliminary screening were downloaded and carefully reviewed, and studies with incomplete data, low quality, or other ineligible features were further excluded; third, dispute resolution, in which inconsistent opinions between the two researchers were resolved through joint discussion, and if an agreement could not be reached, a third senior researcher was invited to make the final decision to determine the final list of included studies. A standardized data extraction form was developed, and the two researchers independently extracted data from the included studies and then cross-checked the extracted data; inconsistent data were verified by rechecking the original articles.

The extracted data included the following items: basic study information including first author, publication year, study region, study type (cohort study / case-control study), and total sample size; baseline characteristics of study participants including diabetes type, age at enrollment or baseline, DR diagnostic method, and follow-up duration; and data related to exposure and outcome factors including risk factors, effect sizes [odds ratio (OR), relative risk (RR), hazard ratio (HR)], number of DR onset cases, and number of DR progression cases.

### 2.5 Study quality assessment

Two researchers independently assessed the quality of the included studies using appropriate quality assessment tools. The assessment process was strictly performed in accordance with the tool instructions, and the results were cross-checked. Discrepancies were resolved through discussion or consultation with a third researcher.

The Newcastle-Ottawa Scale (NOS) (12) was used for quality assessment of cohort studies. This scale consists of three dimensions: selection of study participants (4 points), comparability between groups (2 points), and assessment of outcome (3 points), with a total of 8 items. It adopts a star-based semi-quantitative system with a maximum score of 9 stars. Studies with a score ≥7 stars were defined as high-quality studies, those with 4–6 stars as moderate-quality studies, and those with ≤ 3 stars as low-quality studies. Low-quality studies were excluded.

### 2.6 Statistical analysis

All meta-analyses, sensitivity analyses, and publication bias tests in this study were performed using R software version 4.4.2. Statistical calculations were mainly conducted with the meta, metaphor, and metafor packages. A two-sided test with α = 0.05 was used as the significance level.

#### 2.6.1 Effect size selection and pooling

Appropriate effect sizes were selected based on study design and data type. For case-control studies, OR and 95% 95% CI were used to reflect the strength of association between risk factors and DR onset or progression. For cohort studies, RR or HR with 95% CI was adopted, and HR was used for long-term association analyses adjusted for follow-up time.

When effect sizes for the same risk factor were inconsistent across studies, effect sizes were first standardized and converted to HR before pooling to ensure comparability. Standard conversion methods from published methodological studies were used to convert OR and RR to HR. Meta-analysis was performed for risk factors with ≥5 included studies, and effect sizes were pooled using fixed-effect or random-effect models. For risk factors with fewer than 5 studies, only qualitative description was provided without quantitative pooling to avoid bias due to insufficient sample size.

#### 2.6.2 Heterogeneity test

The Q test and *I*^2^ test were used to evaluate heterogeneity across studies. The Q test provided qualitative assessment: *P* > 0.10 indicated no significant heterogeneity, while *P* ≤ 0.10 indicated significant heterogeneity. The *I*^2^ test provided quantitative evaluation: *I*^2^ < 50% indicated acceptable heterogeneity, while I^2^ ≥ 50% indicated significant heterogeneity.

A fixed-effect model was used for acceptable heterogeneity (*I*^2^ < 50% and *P* > 0.10). A random-effect model was applied for significant heterogeneity (*I*^2^ ≥ 50% or *P* ≤ 0.10), and subgroup analysis and meta-regression were further used to explore sources of heterogeneity.

#### 2.6.3 Sensitivity analysis

A leave-one-out method was used for sensitivity analysis. Each included study was omitted in turn, and meta-analysis was repeated. Changes in pooled effect sizes (OR/RR/HR and 95% CI) before and after omission were compared. A notable change in pooled effect size (e.g., 95% CI crossed 1) after omitting one study indicated that study as a source of heterogeneity. Stable pooled effect sizes after omitting any single study suggested good robustness and reliability of the results.

#### 2.6.4 Publication bias test

Multiple methods were combined to assess publication bias: funnel plot: A symmetric funnel plot indicated no obvious publication bias, while an asymmetric plot suggested potential publication bias. Egger's test: *P* > 0.05 indicated no significant publication bias, and *P* ≤ 0.05 indicated significant publication bias. Trim-and-fill method: If publication bias existed, the trim-and-fill method was used to supplement potentially missing studies, and effect sizes were repooled. Similar pooled effect sizes before and after trimming suggested little impact of publication bias. Obvious changes required careful interpretation and detailed discussion in the text.

## 3 Results

### 3.1 Literature screening results

A systematic search was performed in the PubMed, Web of Science, Embase, and Scopus databases. A total of 4,482 records were initially identified (745 from PubMed, 405 from Web of Science, 1,470 from Embase, and 1,862 from Scopus). After duplicate removal using EndNote, 2,710 records remained for title and abstract screening.

In the preliminary screening stage, 2,523 ineligible records were excluded according to the preset inclusion and exclusion criteria. The main reasons for exclusion were as follows: animal studies (64 records), review articles (347 records), cross-sectional studies (304 records), study populations not consisting of children or adolescents (856 records), topics irrelevant to risk factors for DR (654 records), and studies of special populations with severe ocular or systemic comorbidities (298 records). Finally, 187 records were retained for full-text evaluation.

**Figure 1:**
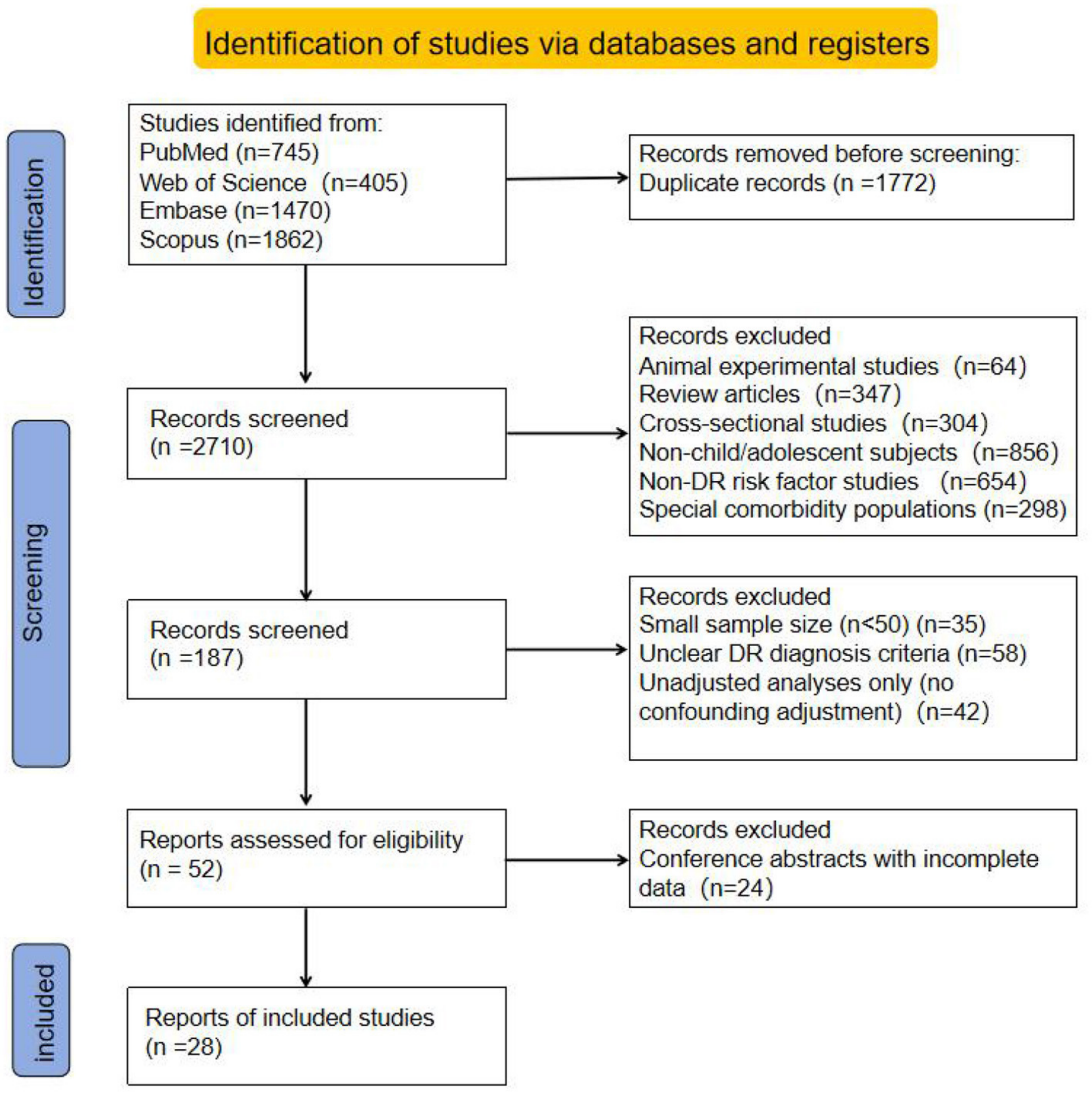
PRISMA flow diagram.

In the full-text secondary screening stage, study design, data completeness, and methodological quality were further assessed. A total of 134 articles were excluded. The main reasons included sample size less than 50 (35 articles), unclear DR diagnostic criteria (58 articles), only unadjusted univariate analysis results reported (42 articles), and conference abstracts with missing key data (24 articles). Methodological quality was assessed during full-text screening, and only studies with moderate or high NOS scores were included. After strict screening, 28 articles ultimately met all inclusion criteria for this meta-analysis and were used for subsequent data extraction and pooled analysis. Details of the literature screening process are shown in Figure 1.

### 3.2 Basic characteristics of included studies

A total of 28 cohort studies were finally included in this study (13–40), covering children and adolescents with diabetes from multiple regions worldwide, with a total sample size of 71,697 participants. The smallest sample size was 130 (35) and the largest was 11,326 (29), showing a balanced distribution and a large overall scale. The study regions included Europe (Sweden, Denmark, Norway, Germany, Austria, Italy), the Americas (the United States, Canada), Oceania (Australia, New Zealand), and Asia (Taiwan, China), involving populations of different races and socioeconomic backgrounds. All included studies clearly defined diabetes type, with type 1 diabetes (T1DM) being predominant (23 studies, accounting for 93.84% of participants). Four studies included both T1DM and type 2 diabetes (T2DM) patients, and one study included only T2DM patients. The age range of participants was 0–25 years, with the median baseline age ranging from 7.1 to 18.4 years, consistent with the definition of children and adolescents. Although our inclusion criterion was age ≤ 20 years, some included studies reported a wider age range (0–25 years or < 30 years). We only extracted data and effect estimates from participants aged ≤ 20 years to ensure strict consistency with the study protocol. Follow-up duration ranged from 1.3 to 40 years, including 10 studies with follow-up ≥10 years and the longest follow-up reaching 40 years. Internationally recognized standards were adopted for DR diagnosis and grading. Fundus photography (dilated/non-dilated) was the main diagnostic method (25 studies). Some studies were assisted by fundoscopy, ICD codes, or surgical records. The main grading systems were the Early Treatment Diabetic Retinopathy Study (ETDRS) grading (8 studies) and modified Airlie House classification (7 studies). The remaining studies used the International Clinical DR Severity Scale, International Society for Pediatric and Adolescent Diabetes (ISPAD) grading, and others. The high standardization of diagnosis and grading reduced the impact of measurement bias on the results. Details are presented in Table 1.

**Table 1:** Basic characteristics of the included studies.

References	Year	Region	Study design	Total sample size	Type of diabetes	Age (Enrollment/Baseline)	DR diagnostic method	Follow–up
Anderzén et al. (13)	2016	Sweden	Retrospective cohort study	4250	T1DM	13–18 years	Fundus photography	NA
Benitez et al. (14)	2022	UK/Canada/Australia	Prospective cohort study	710	T1DM	14.3 ± 1.6 years	Standardized 2–field fundus photography, ETDRS grading	Median 3.2 years
Bonney et al. (15)	1995	Australia	Prospective cohort study	203	T1DM	10.4–20.6 years, median 14.5 years	Stereoscopic fundus photography, modified Airlie House classification	Median 1.3 years
Broe et al. (16)	2014	Denmark	Prospective cohort study	185	T1DM	21.0 ± 3.3 years	Standardized 2–field fundus photography, ETDRS grading	16 years
Cheung et al. (17)	2008	Australia	Prospective cohort study	645	T1DM	12–20 years	7–field stereoscopic fundus photography, modified Airlie House classification	Median 2.5 years
Dabelea et al. (18)	2017	United States	Retrospective cohort study	T1DM:1746, T2DM:272	T1DM and T2DM	10–25 years	45° color digital fundus photography, modified Airlie House grading criteria	Mean 7.9 years
Ek et al. (19)	2020	Sweden	Prospective cohort study	T1DM:3748, T2DM:1413	T1DM and T2DM	10–17.9 years	Fundus photography	5 years
Gallego et al. (20)	2008	Australia	Prospective cohort study	1869	T1DM	13.4 years (interquartile range 12.0–15.2 years)	7–field stereoscopic fundus photography, modified Airlie House classification	Median 4.1 years
Hammes et al. (21)	2011	Germany	Prospective cohort study	8784	T1DM	18–60 years (including adolescent–onset population)	Dilated direct ophthalmoscopy, modified Airlie House classification	Median 1.5 years
Hietala et al. (22)	2012	Finland	Retrospective cohort study	369	T1DM	Median 8.4 years	Dilated ophthalmoscopy and/or fundus photography, ETDRS grading	Median 19.6 years
Januszewski et al. (23)	2022	Australia	Prospective cohort study	2063	T1DM	10–18 years	7–field stereoscopic fundus photography, ETDRS grading	Median 4.1 years
Jensen et al. (24)	2023	United States	Prospective cohort study	T1DM:2519, T2DM:447	T1DM and T2DM	Adolescents and young adults	Non–dilated fundus photography, modified Airlie House classification	Mean 7.5 years
Klein et al. (25)	1997	United States	Prospective cohort study	354	T1DM	≤ 30 years	7–field 30° color stereoscopic fundus photography, Wisconsin DR Study grading	4 years
Krolewski et al. (26)	1986	United States	Prospective cohort study	292	T1DM	0–20 years	Fundus photography, modified Airlie House classification	20–40 years
Levitsky et al. (27)	2022	United States	Prospective cohort study	518	T2DM	10–17 years	Digital fundus photography, ETDRS grading	2–6.5 years
Lind et al. (28)	2019	Sweden	Prospective cohort study	10398	T1DM	Mean 14.7 years	Ophthalmoscopy + fundus photography, ETDRS grading	8–20 years
Liu et al. (29)	2021	Sweden	Prospective cohort study	11326	T1DM	Median 9.6 years	Ophthalmoscopy + fundus photography, ICD code classification	Median 7.5 years
Rasmussen et al. (30)	2015	Denmark	Prospective cohort study	138	T1DM	20.6 ± 3.0 years	Field fundus photography, ETDRS grading	16 years
Rathsman et al. (31)	2021	Sweden	Prospective cohort study	11024	T1DM	≤ 18 years	Ophthalmoscopy + fundus photography, ICD code classification	Median 8.2 years
Salardi et al. (32)	2021	Italy	Retrospective cohort study	243	T1DM	Mean 5.7–5.8 years	Dilated fundus photography, simplified grading of the American Academy of Ophthalmology	Follow–up for 16–17 years
Samuelsson et al. (33)	2014	Sweden	Retrospective cohort study	1543	T1DM	5–19 years (median 13.9 years)	Ophthalmoscopy + fundus photography, ICD code classification	Median 7.1 years
Skrivarhaug et al. (34)	2006	Norway	Prospective cohort study	294	T1DM	Median 8.1 years	Dilated 45° fundus photography, International Clinical DR Severity Scale	Median 24.3 years
Thorsen et al. (35)	2015	Denmark	Prospective cohort study	130	T1DM	Median 7.77 ± 3.70 years	Fundus photography according to EURODIAB protocol, ETDRS grading	16 years
Velayutham et al. (36)	2020	Australia	Prospective cohort study	904	T1DM	Median 14.0 ± 1.5 years	Dilated 7–field stereoscopic fundus photography, International Clinical DR grading	Median 3 years
Wang et al. (37)	2016	China	Retrospective cohort study	153	T1DM	Median 7.0 ± 4.0 years	Dilated 45° fundus photography, ETDRS	Mean 13.1 ± 2.7 years
Wang et al. (38)	2017	United States	Retrospective cohort study	T1DM:2240, T2DM:1768	T1DM and T2DM	≤ 21 years	ICD−9–CM diagnostic codes	Median 3.2 years
White et al. (39)	2017	Australia	Retrospective cohort study	503	T1DM	Mean 18.4 years	Ophthalmoscopy + photocoagulation surgery records	Mean 8.5 years
Winter et al. (40)	2024	New Zealand	Retrospective cohort study	646	T1DM	7.4 ± 3.6 years	Fundus photography, ISPAD grading	15 years

Abbreviations in the text are defined as follows: DR, Diabetes Retinopathy; ETDRS, Early Treatment Diabetic Retinopathy Study; ICD, International Classification of Diseases; ISPAD, International Society for Pediatric and Adolescent Diabetes; EURODIAB, European Diabetes Epidemiology Study Group; NA, not available.

### 3.3 Summary of risk factors and outcome measures in included studies

The 28 cohort studies included in this study reported more than 20 risk factors associated with the onset and progression of DR in children and adolescents. These risk factors fell into five categories: metabolic indicators, disease characteristics, physiological parameters, demographic characteristics, and other potentially associated factors. All risk factors were analyzed after multivariable adjustment, with effect sizes including OR, HR, and RR. Details are shown in Table 2. Regarding outcome measures, 17 studies focused on DR onset (only reporting incident DR cases), 1 study focused on DR progression (only reporting cases of upgraded DR severity), and 5 studies investigated both DR onset and progression. The number of DR onset cases ranged from 16 to 3,462, and the number of DR progression cases ranged from 23 to 703.

**Table 2:** Summary of risk factors and outcome indicators related to DR in children and adolescents.

References	Year	Baseline DR status of study subjects	DR progression criteria	Risk factor	Study outcome	Effect size	Number of incident DR cases	Number of progressive DR cases
Anderzén et al. (13)	2016	No DR record at baseline	NA	HbA1c	DR incidence	OR	1843	NA
Benitez et al. (14)	2022	No definite severe DR at baseline	≥1–step increase in ETDRS grade	Urinary albumin/creatinine ratio, HbA1c, DBP	DR progression	HR	NA	83
Bonney et al. (15)	1995	41% with DR and 59% without DR	≥1–step increase in modified Airlie House grade	DD, HbA1c	DR incidence + progression	OR	32	23
Broe et al. (16)	2014	44.9% without DR, 54.6% with non–proliferative DR, 0.5% with proliferative DR	NA	CRAE, CRVE, HbA1c, DBP, age, sex	DR incidence	OR	50	NA
Cheung et al. (17)	2008	No DR at baseline	NA	CRAE, CRVE	DR incidence	HR	274	NA
Dabelea et al. (18)	2017	No DR record at baseline	NA	Type of diabetes, waist–to–height ratio, MAP	DR incidence	OR	107	NA
Ek et al. (19)	2020	No DR record at baseline	NA	Type of diabetes, sex, BMI, HbA1c, SBP, DBP	DR incidence	HR	1216	NA
Gallego et al. (20)	2008	No DR at baseline	NA	SBP, DBP, HbA1c, DD, BMI, sex, age	DR incidence	HR	673	32
Hammes et al. (21)	2011	No DR record at baseline	≥1–step increase in modified Airlie House grade	age, HbA1c, smoking, sex, SBP, DBP, dyslipidemia	DR incidence + DR progression	OR	2407	703
Hietala et al. (22)	2012	No DR record at baseline	NA	HbA1c, MAP, age at onset	DR incidence	HR	115	NA
Januszewski et al. (23)	2022	No DR or early DR at baseline	≥1–step increase in ETDRS grade	HbA1c, DD, age	DR incidence, DR progression	HR	84	194
Jensen et al. (24)	2023	No DR at baseline	≥1–step increase in modified Airlie House grade	HbA1c, SBP, DBP, BMI z–score, waist–to–height ratio	DR incidence, DR progression	RR	1542	555
Klein et al. (25)	1997	No DR at baseline	NA	HbA1c, age, SBP, DBP	DR incidence	OR	18	NA
Krolewski et al. (26)	1986	No DR at baseline	NA	Hyperglycemia frequency index, age at diabetes onset	DR incidence	RR	99	NA
Levitsky et al. (27)	2022	No DR at baseline	NA	BMI	DR incidence	OR	71	NA
Lind et al. (28)	2019	No DR at baseline	NA	HbA1c, age at onset	DR incidence	OR	3462	NA
Liu et al. (29)	2021	No DR at baseline	NA	Neurodevelopmental disorders (ADHD, autism, intellectual disability), HbA1c	DR incidence	HR	2628	NA
Rasmussen et al. (30)	2015	No DR at baseline	Progression to severe NPDR/PDR	MA count, HbA1c	DR incidence + progression	OR	30	73
Rathsman et al. (31)	2021	No DR at baseline	NA	LDL, cholesterol, HbA1c, age at onset, sex, BMI, smoking	DR incidence	HR	4147	NA
Salardi et al. (32)	2021	No DR at baseline	NA	HbA1c, age at onset	DR incidence	OR	79	NA
Samuelsson et al. (33)	2014	No DR at baseline	NA	HbA1c, smoking	DR incidence	OR	262	NA
Skrivarhaug et al. (34)	2006	Baseline: 194 cases without DR, 97 cases with NPDR, 3 cases with PDR	Progression to PDR	HbA1c, TG, sex	DR progression + incidence	RR	107	67
Thorsen et al. (35)	2015	No DR or mild DR at baseline	≥1–step increase in ETDRS grade	Genetic polymorphisms (CTSH/rs3825932, ERBB3/rs2292239)	DR progression + PDR incidence	OR	21	52
Velayutham et al. (36)	2020	No DR at baseline	NA	MWa, MWv	DR incidence	OR	181	NA
Wang et al. (37)	2016	No DR at baseline	NA	age at onset, TG, HbA1c	DR incidence	HR	25	NA
Wang et al. (38)	2017	No DR at baseline	NA	Type of diabetes, HbA1c, age at onset, sex, household net worth	DR incidence	HR	578	NA
White et al. (39)	2017	No DR at baseline	NA	HbA1c, DD	DR incidence	OR	16	NA
Winter et al. (40)	2024	No DR at baseline	NA	age at onset, DD, HbA1c, race	DR incidence	RR	359	NA

HbA1c, glycated hemoglobin; DD, diabetes duration; CRAE, central retinal artery equivalent; CRVE, central retinal vein equivalent; DBP, diastolic blood pressure; SBP, systolic blood pressure; BMI, body mass index; MAP, mean arterial pressure; hsCRP, high–sensitivity C–reactive protein; TNF–R1/2, tumor necrosis factor receptor 1/2; LDL, low–density lipoprotein; TG, triglyceride; MA, microaneurysm; NPDR, non–proliferative diabetic retinopathy; PDR, proliferative diabetic retinopathy; ADHD, attention deficit hyperactivity disorder; MWa, mean width of arterioles in the extended region; MWv, mean width of venules in the extended region; OR, odds ratio; HR, hazard ratio; RR, relative risk; NA, not available.

In terms of risk factor distribution, HbA1c was the most extensively investigated core factor, with 22 studies analyzing it. All these studies confirmed a significant positive correlation between HbA1c and DR onset or progression. Traditional classic risk factors included diabetes duration (5 studies), age at onset (13 studies), blood pressure (systolic/diastolic blood pressure, 7 studies), blood lipids (total cholesterol/triglycerides/low-density lipoprotein, 4 studies), and body mass index (BMI, 5 studies), with consistent results across multiple studies. Emerging or specific risk factors, such as retinal vascular parameters (central retinal artery equivalent [CRAE], central retinal vein equivalent [CRVE], etc., 2 studies), race (1 study), neurodevelopmental disorders (1 study), and gene polymorphism (1 study), were only reported in a small number of studies, and their associations require further verification.

### 3.4 Methodological quality assessment of included studies

The overall NOS score of eligible studies ranged from 6 to 9 points, and no low-quality literature was included in the final meta-analysis. Specifically, 24 studies were rated as high quality (NOS score: 7-9 points), and 4 studies were graded as moderate quality (NOS score: 6 points).No low-quality studies were included. Details are presented in Table 3.

**Table 3:** Newcastle–Ottawa scale quality assessment.

References	Selection (*n*/4)	Comparability (*n*/2)	Outcome (*n*/3)	Total (*n*/9)	Quality grade
Anderzén et al. (13)	3	2	2	7	High
Benitez et al. (14)	4	2	2	8	High
Bonney et al. (15)	3	1	2	6	Moderate
Broe et al. (16)	3	2	2	7	High
Cheung et al. (17)	4	2	2	8	High
Dabelea et al. (18)	3	2	2	7	High
Ek et al. (19)	4	2	2	8	High
Gallego et al. (20)	3	2	2	7	High
Hammes et al. (21)	3	2	2	7	High
Hietala et al. (22)	4	2	2	8	High
Januszewski et al. (23)	4	2	2	8	High
Jensen et al. (24)	4	2	2	8	High
Klein et al. (25)	3	1	2	6	Moderate
Krolewski et al. (26)	3	2	2	7	High
Levitsky et al. (27)	3	2	2	7	High
Lind et al. (28)	4	2	2	8	High
Liu et al. (29)	4	2	2	8	High
Rasmussen et al. (30)	3	2	2	7	High
Rathsman et al. (31)	4	2	2	8	High
Salardi et al. (32)	3	1	2	6	Moderate
Samuelsson et al. (33)	3	2	2	7	High
Skrivarhaug et al. (34)	4	2	2	8	High
Thorsen et al. (35)	3	1	2	6	Moderate
Velayutham et al. (36)	3	2	2	7	High
Wang et al. (37)	3	2	2	7	High
Wang et al. (38)	4	2	2	8	High
White et al. (39)	3	2	2	7	High
Winter et al. (40)	4	2	2	8	High

NOS scoring was performed strictly based on cohort study criteria.Quality grading: High (7–9 stars), Moderate (4–6 stars), Low ( ≤ 3 stars).

### 3.5 Pooled effect sizes of risk factors for DR onset and progression

This study performed a systematic evaluation and meta-analysis of the associations between various risk factors and the risk of DR onset and progression based on the preset inclusion and exclusion criteria. Risk factors with fewer than 5 included studies were not pooled to ensure the reliability and statistical power of the results. The final risk factors included in the analysis were glycated hemoglobin (HbA1c) (13, 15, 16, 19–26, 28–34, 37–40), diastolic blood pressure (DBP) (16, 19–21, 24, 25), systolic blood pressure (SBP) (19–21, 24, 25), diabetes duration (15, 20, 23, 39, 40), BMI (19, 20, 24, 27, 31), sex (16, 19–21, 31, 34, 38), and age at diabetes onset (16, 20–23, 25, 26, 28, 31, 32, 37, 38, 40), with 5 to 22 studies included for each factor.

Pooled effect size results are presented in Table 4. Given the high between-study heterogeneity (*I*^2^ > 75% for most outcomes), the random-effects model was chosen as the primary analysis model. to account for inter-study variability. In the random-effects model, elevated HbA1c was significantly associated with an increased risk of both DR onset (HR = 1.76, 95% CI: 1.45–2.14) and DR progression (HR = 1.65, 95% CI: 1.15–2.37). The 95% confidence intervals for these two associations did not include 1, indicating statistical significance (*P* < 0.05).

**Table 4:** Pooled effect sizes of each risk factor for the occurrence and progression of DR.

Risk factor	Outcome	Fixed–effects model HR (95%)	Random–effects model HR (95%)	*I* ^2^	Heterogeneity *P*–value	Number of study samples
HbA1c	Risk of DR incidence	1.02 (1.02–1.02)	1.76(1.45–2.14)	98.0%	< 0.001	22
HbA1c	Risk of DR progression	1.04 (1.02–1.05)	1.65(1.15–2.37)	92.6%	< 0.001	7
DBP	Risk of DR incidence	1.00 (0.99–1.00)	1.22(0.98–1.53)	89.8%	< 0.001	6
SBP	Risk of DR incidence	1.01 (1.01–1.02)	1.17(0.94–1.45)	82.3%	< 0.001	5
Diabetes duration	Risk of DR incidence	1.16 (1.13–1.19)	1.38(0.93–2.06)	83.3%	< 0.001	5
BMI	Risk of DR incidence	1.01 (1.00–1.02)	1.03(0.81–1.31)	77.9%	0.001	5
Sex (male vs female)	Risk of DR incidence	1.03 (0.98–1.08)	1.36(0.97–1.90)	89.9%	< 0.001	7
Age at onset	Risk of DR incidence	0.93 (0.92–0.95)	0.84(0.62–1.15)	98.1%	< 0.001	13

HbA1c, glycated hemoglobin; DBP, diastolic blood pressure; SBP, systolic blood pressure; BMI, body mass index; DR, diabetic retinopathy; HR, hazard ratio. The p-values presented in this table are derived from the Cochran's Q test for heterogeneity, not from the significance test of the pooled effect sizes. Significance of each pooled HR was determined by whether the 95% confidence interval crossed 1.

For other risk factors (including blood pressure, diabetes duration, BMI, and sex), the pooled 95% CIs all crossed 1, indicating no statistically significant associations with DR onset in the random-effects model. The results from the fixed-effects model are provided in the table for completeness but are not interpreted due to the high heterogeneity.

### 3.6 Analysis of sources of between-study heterogeneity and sensitivity analysis

Leave-one-out sensitivity analysis indicated that the pooled results of three risk factors (SBP, diabetes duration, and BMI) showed relatively weak stability, and the core sources of heterogeneity were identified. The overall *I*^2^ for systolic blood pressure was 82.3%, which decreased significantly to 67.6% after excluding the study by Hammes et al. (21). The overall *I*^2^ for diabetes duration was 83.3%, which dropped notably to 60.1% after excluding the study by Bonney et al. (15). The overall *I*^2^ for BMI was 77.9%, which decreased to 66.7% after excluding the study by Levitsky et al. (27). These results show that heterogeneity in these three factors was driven partly by outlier studies, though moderate-to-high heterogeneity remained.

In contrast, the direction and overall trend of the pooled effect sizes for HbA1c, diastolic blood pressure, age at diabetes onset, and sex remained stable, with no substantial reversal after excluding any single study. The pooled HR for HbA1c was 1.76 (95% CI: 1.45–2.14, *I*^2^ = 98.0%), and the *I*^2^ remained between 96.5% and 98.12% after exclusion. The pooled HR for diastolic blood pressure was 1.22 (95% CI: 0.98–1.53, *I*^2^ = 89.8%), with the *I*^2^ ranging from 83.0% to 91.2% after exclusion. The pooled HR for age at diabetes onset was 0.8421 (95% CI: 0.6181–1.1473, *I*^2^ = 98.1%), and the *I*^2^ remained as high as 96.5% to 98.3% after exclusion. The pooled HR for sex was 1.3586 (95% CI: 0.9693–1.9043, *I*^2^=89.9%), with the *I*^2^ maintained at 88.4% to 91.0% after exclusion. Heterogeneity of these indicators was not significantly reduced by excluding a single study, indicating that the sources of heterogeneity could not be explained by individual studies.

Subgroup and meta-regression analyses were only performed for the four factors whose heterogeneity could not be explained by outlier studies. For SBP, diabetes duration, and BMI, no further subgroup analysis was done because heterogeneity was driven mainly by individual outlier studies rather than general clinical or methodological differences. Although significant between-subgroup differences were found in some analyses, high heterogeneity still remained within subgroups. Significant subgroup differences only indicate partial explanation of heterogeneity, not full resolution.

**Figure 2:**
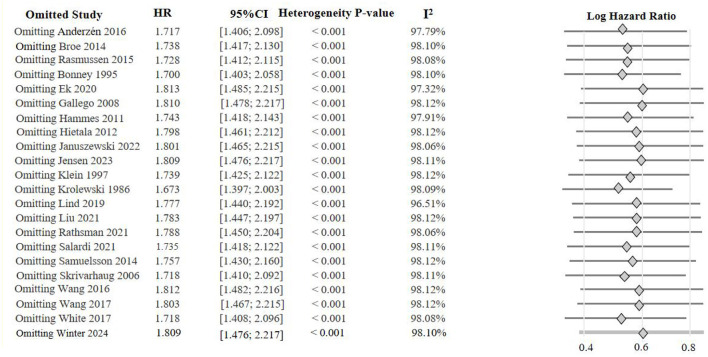
Leave-one-out sensitivity analysis of HbA1c-associated diabetic retinopathy onset.

Leave-one-out sensitivity analysis for the primary outcome (HbA1c and DR onset) is presented in Figure 2.

#### 3.6.1 Potential sources of heterogeneity in the association between HbA1c and DR

To explore potential sources of heterogeneity in the association between HbA1c and DR onset risk, random-effects meta-regression analysis was performed using the restricted maximum likelihood (REML) method based on data from 22 included studies. The overall model was highly significant (Q_m_ = 30.62, *P* < 0.001) and explained 61.43% of heterogeneity (R^2^ = 61.43%). The results indicated that exposure metric (*P* < 0.001) and sample size (*P* < 0.001) were the main sources of heterogeneity in the association between HbA1c and DR onset risk. Compared with the “high vs. Low” dichotomous comparison, effect sizes for “per 1% increase in HbA1c” were significantly lower (95% CI:−1.27 to−0.44). Larger sample sizes were associated with significantly reduced effect sizes (95% CI:−0.43 to−0.15). Study design, region, diabetes type, and follow-up duration had no significant impact on the association strength (all *P* > 0.05), suggesting good robustness of the results.

**Figure 3:**
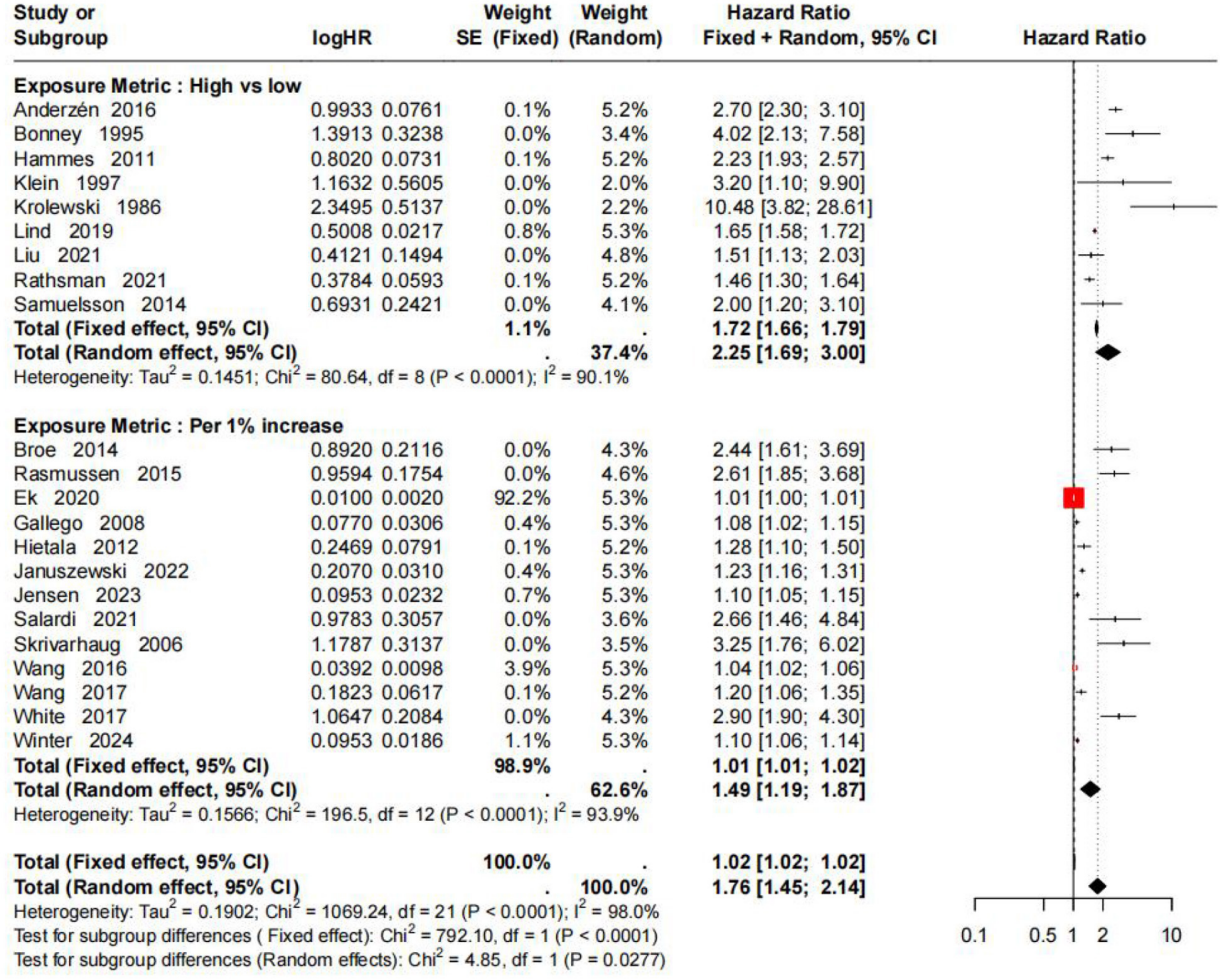
Subgroup analysis of DR risk based on different HbA1c exposure metrics.

Subgroup analysis stratified by HbA1c exposure metric showed that the pooled HR for the high HbA1c vs. low HbA1c group was 2.25 (95% CI: 1.69–3.00, *I*^2^ = 90.1%), and the pooled HR for the per 1% increase in HbA1c group was 1.52 (95% CI: 1.22–1.89, *I*^2^ =93.3%). The between-subgroup difference was statistically significant (random-effects model: χ^2^ = 4.49, *P* = 0.034), indicating that exposure metric was an important source of heterogeneity. Details are shown in Figure 3.

**Figure 4:**
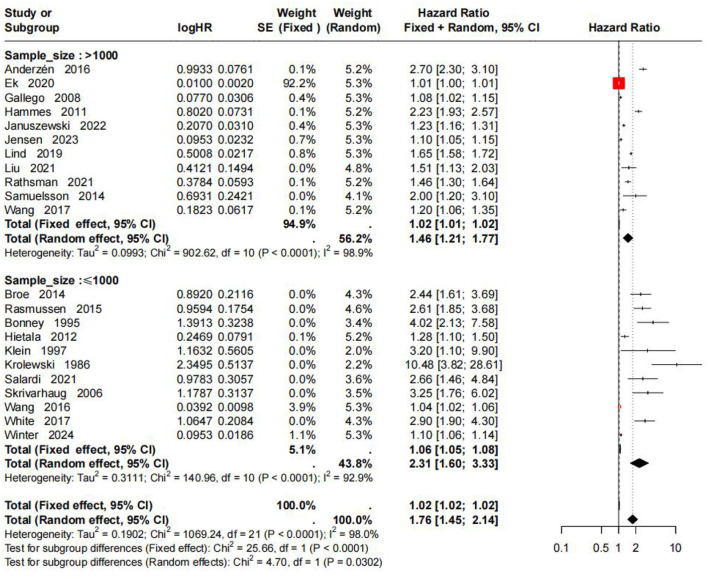
Subgroup analysis of DR risk stratified by study sample size.

Subgroup analysis by sample size showed that the pooled HR for the large-sample group (>1,000 participants) was 1.46 (95% CI: 1.21–1.77, *I*^2^ = 98.9%), and the pooled HR for the small-sample group ( ≤ 1,000 participants) was 2.31 (95% CI: 1.60–3.33, *I*^2^ = 92.9%). Between-subgroup differences were significant in both the fixed-effects model (χ^2^ = 25.66, *P* < 0.0001) and the random-effects model (χ^2^ = 4.70, *P* = 0.0302), suggesting that sample size was an important source of heterogeneity. Small-sample studies tended to overestimate the strength of the association between HbA1c and DR onset risk. Details are shown in Figure 4.

For the association between HbA1c and DR progression risk, 7 cohort studies were included in the meta-analysis. Leave-one-out sensitivity analysis showed that the pooled HR from the random-effects model remained stable between 1.47 and 1.80 after excluding any single study. The I^2^ statistic consistently ranged from 90.3% to 93.7%, which was highly consistent with the overall analysis (HR =1.65, 95%CI: 1.15–2.37, *P* = 0.0067, *I*^2^ = 92.6%). These findings indicate robust and reliable results that were not excessively influenced by any single study. Univariate subgroup analysis revealed no statistically significant between-group differences, suggesting that between-study heterogeneity may be driven by multiple intermingled factors that cannot be fully identified by single-dimensional stratification. Further multivariate meta-regression analysis showed that after adjusting for exposure metric, sample size, and region, the overall model adjustment test was highly significant (Q_m_ = 24.02, *P* < 0.0001) and explained 88.20% of between-study heterogeneity (R^2^ = 88.20%). Exposure metric (*P* = 0.0008), sample size (*P* = 0.0448), and region (*P* = 0.0016) were independent significant sources of heterogeneity.

#### 3.6.2 Potential sources of heterogeneity in the association between DBP and DR

**Figure 5:**
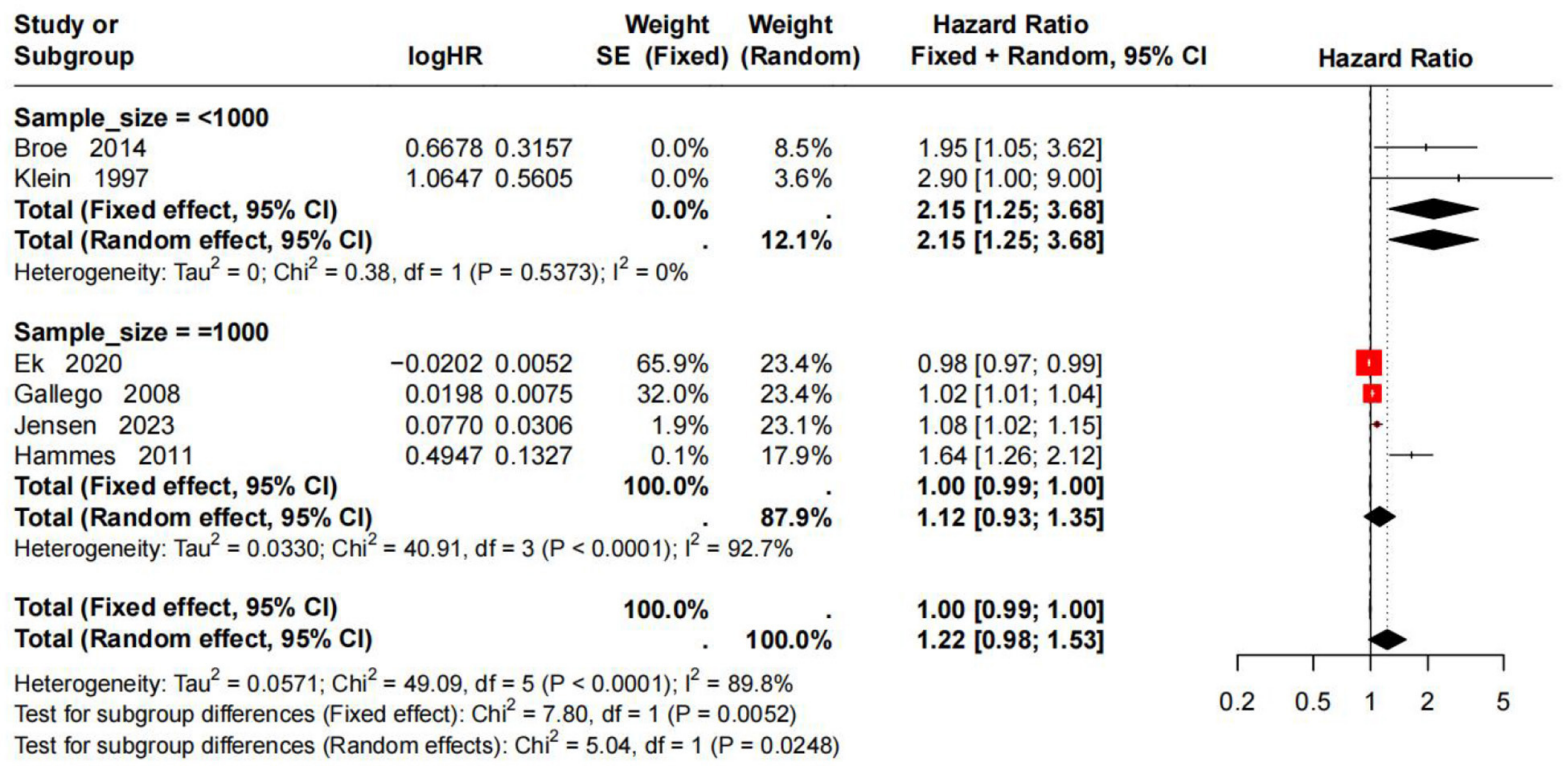
Subgroup analysis of DBP and the risk of DR (grouped by sample size).

Subgroup analysis of diastolic blood pressure and DR onset risk stratified by sample size showed that the pooled HR was 2.15 (95%CI: 1.25–3.68, *I*^2^ =0%) for the small-sample group (sample size < 1,000) and 1.12 (95%CI: 0.93–1.35, *I*^2^ =92.7%) for the large-sample group (sample size ≥ 1,000). Between-subgroup differences were highly significant in the fixed-effect model (χ^2^=7.80, *P* = 0.0052) and significant in the random-effect model (χ^2^ = 5.04, *P* = 0.0248), indicating that sample size was an important source of heterogeneity. Small-sample studies tended to overestimate the strength of the association between diastolic blood pressure and DR. Details are presented in Figure 5.

#### 3.6.3 Potential sources of heterogeneity in the association between age at diabetes onset and DR

Variables including exposure metric, study design, sample size, region, and follow-up duration were included in the meta-regression analysis. The overall model was not statistically significant (Q_m_ = 8.98, *P* = 0.1747) and only explained 18.38% of the heterogeneity. None of the subgroup factors showed a significant moderating effect (all *P* > 0.05), indicating that the high heterogeneity in this study could not be fully explained by the analyzed variables.

#### 3.6.4 Potential sources of heterogeneity in the association between sex (Male vs. Female) and DR

Univariate subgroup analysis did not identify any statistically significant sources of heterogeneity (all *P* > 0.05). Further multivariate meta-regression analysis showed that the overall model was highly significant (Q_m_ = 59.07, *P* < 0.001) and explained all of the heterogeneity (R^2^ = 100.00%). Diabetes type, study design, sample size, region, and follow-up duration all exhibited significant moderating effects (all *P* < 0.05). Of note, the regression model may be at risk of overfitting due to the small number of included studies (k = 7), and the conclusions require validation in larger sample studies.

### 3.7 Publication bias

In this study, funnel plots were not used for visual assessment of publication bias for some risk factors because the number of included studies was small (k < 10), leading to insufficient statistical power. Only Egger's test was used for quantitative analysis.

**Figure 6:**
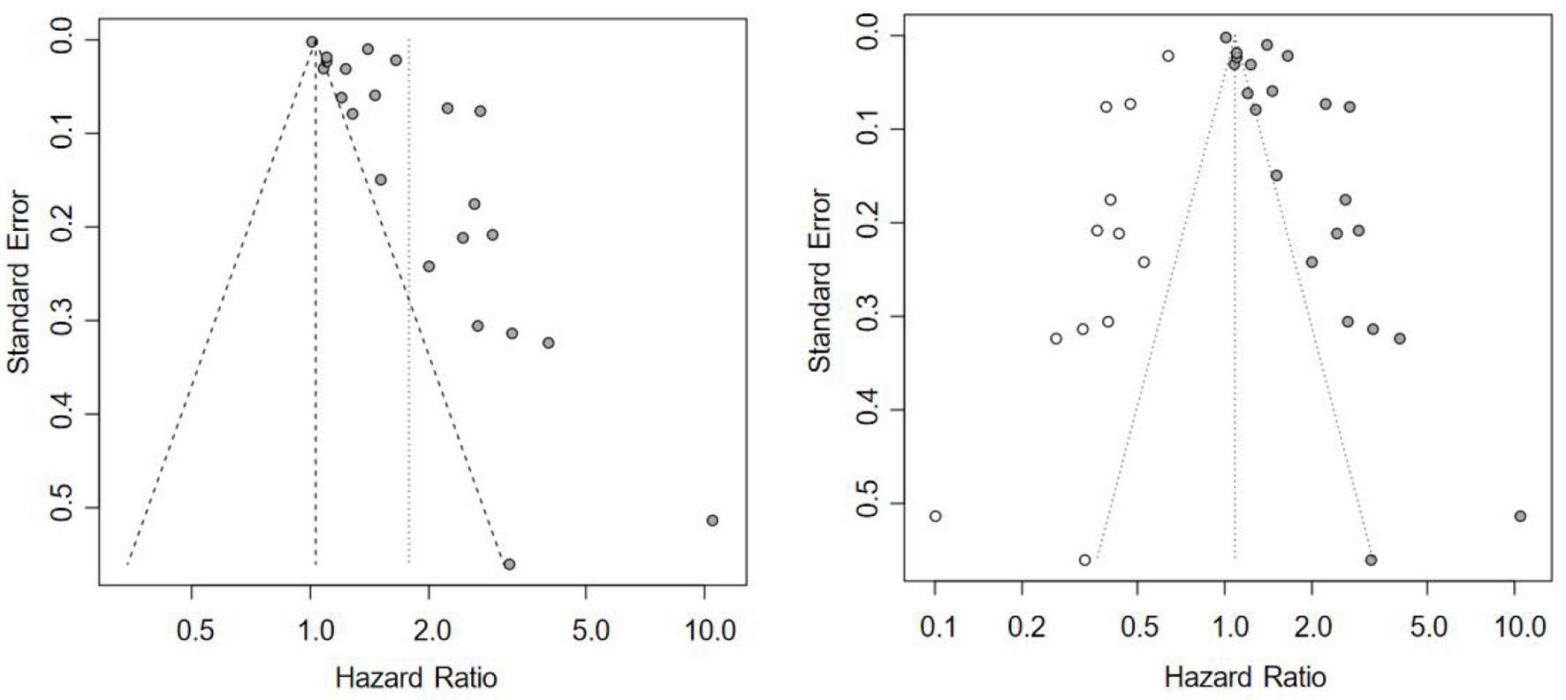
Funnel plot of the association analysis between HbA1c and the risk of DR incidence: before trim-and-fill **(left)** and after trim-and-fill **(right)**.

The results showed varying degrees of publication bias for several indicators. Significant publication bias was detected in the analyses of HbA1c with both DR onset and progression risk (onset risk: *t* = 3.76, *P* = 0.0012; progression risk: *t* = 6.51, *P* = 0.0013). After adjustment by the trim-and-fill method, 12 and 4 missing negative studies needed to be supplemented, respectively. The adjusted pooled effect sizes were not statistically significant (onset risk: HR = 1.09, *P* = 0.5955; progression risk: HR = 1.0509, *P* = 0.8384), indicating that the original positive results were greatly affected by publication bias (see Figure 6). Mild and significant publication bias was also found in the analyses of diastolic blood pressure and systolic blood pressure, respectively (diastolic blood pressure: *t* = 2.87, *P* = 0.0453; systolic blood pressure: *t* = 7.38, *P* = 0.0051). After trim-and-fill adjustment, 3 and 2 studies needed to be supplemented, respectively. The adjusted pooled effect sizes remained non-significant, consistent with the original results, suggesting that the conclusions were robust and reliable.

Publication bias analyses for the other risk factors were favorable. Egger's test showed no significant publication bias for diabetes duration, BMI, sex, or age at diabetes onset (duration: *t* =0.90, *P* = 0.4337; BMI: *t* = 0.37, *P* = 0.7387; sex: *t* = 1.94, *P* = 0.1103; age at onset: *t* = −0.78, *P* = 0.4527), indicating that the results for these indicators were authentic and reliable without obvious influence from publication bias.

## 4 Discussion

This study conducted a systematic review and meta-analysis to comprehensively investigate risk factors for DR onset and progression in children and adolescents. A total of 28 high-quality cohort studies were included, with a combined sample size of 71,697 participants covering populations from Europe, the Americas, Oceania, Asia, and other regions worldwide. These findings provide large-sample, multi-center, high-quality evidence for early screening, risk stratification, and clinical prevention and control of DR in this population.

The results showed that elevated HbA1c was a key associated indicator for the onset and progression of DR in children and adolescents. In the random-effects model, each 1% increase in HbA1c was associated with a 76% increased risk of DR onset and a 65% increased risk of progression. This association was not excessively influenced by any single study, showing good robustness. Traditional risk factors including elevated systolic blood pressure, longer diabetes duration, and younger age at diabetes onset were significantly associated with DR onset risk in the fixed-effects model, but their pooled effect sizes were not statistically significant in the random-effects model, indicating their association strength was greatly affected by study design, baseline population characteristics, and confounding adjustment strategies. Subgroup analysis, multivariate meta-regression, and comprehensive publication bias tests identified exposure metric, sample size, and study region as the main drivers of heterogeneity in the HbA1c-DR association. Notably, the original positive associations between HbA1c and DR onset/progression were significantly affected by publication bias, and their true association strength requires verification by larger-sample, higher-quality prospective cohort studies.

As a core indicator reflecting long-term glycemic control, HbA1c showed a relatively consistent trend of association with the onset and progression of DR in analyses that did not adjust for publication bias. This finding is generally consistent with those of meta-analyses on DR in adults. (41), further confirming that hyperglycemia is the central pathological basis of diabetic microangiopathy and the initiating factor in the development and progression of DR (42).

Compared with adult patients with diabetes, children and adolescents have longer diabetes duration, earlier exposure to hyperglycemia, and immature retinal microvascular development. Poor long-term glycemic control persistently damages the structure and function of retinal microvascular endothelial cells through multiple pathways, including activation of the polyol pathway, accumulation of advanced glycation end products (AGEs), oxidative stress, and the protein kinase C pathway, ultimately increasing the risk of DR onset and progression (43, 44). This study found that the exposure metric of HbA1c significantly affected effect size estimation. The pooled effect size for the “high vs. low” dichotomous comparison was significantly higher than that for the continuous variable analysis of “ per 1% increase”, which is consistent with findings from a Swedish national diabetes registry study (28) and an 11-country multicenter study (45), suggesting a threshold effect between blood glucose and DR risk. Lind et al. (28) demonstrated that the risk of any DR was significantly higher in the HbA1c 7.0%−7.4% group than in the 6.5%−6.9% group (OR = 1.31, *P* = 0.02). The risk of proliferative DR increased exponentially when HbA1c exceeded 8.6% (OR = 5.98, *P* < 0.001). Moreover, the HbA1c < 6.5% group showed no additional benefit but carried an increased risk of hypoglycemia (RR = 1.34, *P* = 0.005). A large-sample study of 156,090 participants by Bratina et al. (45) further validated that each 1 mmol/mol increase in HbA1c was associated with a 3% higher DR risk (OR = 1.03, *P* < 0.0001). Dichotomous grouping (e.g., ≥7.5% vs. < 7.5%) better highlighted the threshold effect, with a significantly higher DR detection rate in the high HbA1c group, confirming a non-linear association. Accordingly, the 2,024 ISPAD clinical practice guideline recommends a routine HbA1c target of ≤ 7.0% for children and adolescents with T1DM, which may be tightened to ≤ 6.5% only when hypoglycemia risk is controllable. The core principle is to balance DR prevention and control with hypoglycemia safety (46). Notably, after adjusting for publication bias in this study, the association between HbA1c and DR was no longer statistically significant. This suggests the above conclusions should be interpreted with caution.

In this study, traditional risk factors including blood pressure, diabetes duration and age at onset did not exhibit stable statistical associations, which differs somewhat from previous research. The core reasons are closely related to the clinical characteristics of pediatric and adolescent diabetes populations and methodological heterogeneity across studies (17, 47, 48). On the one hand, T1DM predominates in children and adolescents with diabetes. Among the 28 included studies, 23 focused on T1DM patients. Compared with adult patients with T2DM, pediatric T1DM patients have a significantly lower incidence of metabolic comorbidities such as abnormal blood pressure, obesity and dyslipidemia. Moreover, most patients are in the early disease stage, with insufficient manifestation of vascular endothelial injury and microvascular complications, which may dilute the effect sizes of blood pressure, BMI and other factors, making it difficult to detect significant statistical associations (49, 50). Multiple global studies on the burden of pediatric and adolescent diabetes have demonstrated that microvascular complications in pediatric T1DM patients mostly emerge after 10 years of disease duration, and the incidence of DR in patients with a disease duration of less than 5 years is below 5%, which also accounts for the unstable association between diabetes duration and DR in this study (51). On the other hand, significant heterogeneity exists across studies in the definition, measurement methods of risk factors and adjustment strategies for confounding factors. For instance, some studies adopted only a single cross-sectional blood pressure measurement rather than the mean blood pressure from long-term follow-up; some failed to adequately adjust for core confounders such as HbA1c and diabetes duration; some diagnosed DR merely with ophthalmoscopy instead of the more accurate dilated fundus photography. These methodological discrepancies jointly contribute to high heterogeneity among studies and affect the pooled effect sizes. In addition, this study identified a significant correlation between younger age at diabetes onset and elevated DR risk in the fixed-effects model, which is highly consistent with the classic findings of the FinnDiane Study Group. The study reported that T1DM patients with onset at 5–14 years had a significantly higher risk of proliferative DR than those with onset after 15 years, indicating that younger onset age leads to longer exposure of retinal microvasculature to abnormal metabolic environments such as hyperglycemia, more remarkable cumulative microvascular damage, and higher DR risk (52). However, the association of age was not statistically significant in the random-effects model. Therefore, it cannot yet serve as definitive evidence for stratified DR screening strategies in children and adolescents. Further high-quality studies are needed for verification.

In this study, multivariate meta-regression was performed to thoroughly explore the sources of heterogeneity across studies. The results showed that 61.43% of the heterogeneity in the association between HbA1c and DR progression risk could be jointly explained by exposure measurement method and sample size. Notably, a larger sample size was associated with a smaller effect size of the HbA1c-DR association, which is consistent with the classical methodological conclusions of meta-analyses. Small-sample studies are prone to overestimating effect sizes due to sampling error and imbalanced baseline characteristics of the study population, whereas large-sample studies can better dilute random errors and balance the distribution of confounding factors, leading to effect size estimates closer to the true association strength (53). For risk factors such as diastolic blood pressure and sex, sensitivity analyses identified the key studies driving heterogeneity, providing specific guidance for quality control and optimized design in future relevant research. However, the heterogeneity in the association between age at diabetes onset and DR was not fully explained by the included variables, suggesting the presence of unmeasured potential confounders, including retinal vascular parameters, genetic polymorphisms, neurodevelopmental disorders, and insulin regimens. These factors have been verified to correlate with the occurrence and progression of DR in several pediatric DR studies, yet quantitative analysis could not be conducted owing to the limited number of relevant literature included in this study, which represents one of the major limitations of this research.

The comprehensive test results for publication bias provide an important reference for interpreting the findings of this study. After the trim-and-fill correction, the pooled effect sizes for the association between HbA1c and the onset and progression of DR in children and adolescents were no longer statistically significant. This suggests that the original positive results were largely influenced by publication bias. As clearly stated in the Cochrane Handbook for Systematic Reviews, the probability of publication for positive results is significantly higher than that for negative results when studies involve long-term follow-up, high investment, or special populations (e.g., children and patients with rare diseases) (54). In a previous similar study, Osinga et al. (55) observed marked asymmetry in the funnel plot for overt hyperthyroidism (*P* = 0.049) in an individual participant data meta-analysis of the association between gestational thyroid function and gestational diabetes mellitus (n = 63,548), indicating publication bias. Another meta-analysis of the global prevalence of DR in pediatric patients with type 2 diabetes reported widespread publication bias (56). Such selective publication overrepresents positive findings and overestimates the true effects of risk factors, which largely explains the non-significant associations after trim-and-fill correction. For DBP and SBP, effect sizes remained non-significant after correction and were consistent with original results, indicating weak genuine correlations with pediatric DR rather than publication bias. No obvious publication bias was found in diabetes duration, BMI and gender. These results are robust and can serve as reliable evidence for clinical practice.

This study has several limitations. This study has several limitations. First, all included cohort studies were of moderate or higher quality. However, inconsistent follow-up duration, unified definition of risk factors and inadequate adjustment for confounders may still introduce bias. Second, extremely high between-study heterogeneity existed across all risk factor analyses, with the maximum I^2^ up to 98%, which may reduce the stability of pooled results. Third, the association between HbA1c and DR turned non-significant after publication bias correction. The original positive findings may be affected by publication bias, and the actual effect should be interpreted cautiously. Fourth, this analysis mainly focused on traditional metabolic and demographic indicators. Emerging risk factors such as retinal vascular parameters and genetic polymorphisms were rarely reported, so a comprehensive risk profile could not be summarized. Fifth, several risk factors included limited studies. Meta-regression may face overfitting, limiting the generalizability of conclusions. Finally, only English articles were included in this review. Language bias may exist, as high-quality studies published in other languages were not covered.

Based on our results and the prevention needs of childhood and adolescent DR, targeted public health measures can be formulated. It is necessary to optimize the comprehensive management system for pediatric diabetes, integrate regular DR screening into daily clinical management, and strengthen the screening capacity of primary medical institutions. Continuous coordination of screening, diagnosis and intervention should be guaranteed. Special attention shall be given to children in rural, remote and low-income families to narrow the gap in medical resource distribution. Health education should be widely carried out to raise the awareness of parents and clinicians regarding pediatric DR. Emphasis on early control of blood glucose and blood pressure helps form standardized prevention and management awareness. Building a specialized database for pediatric diabetes and DR is also essential. Long-term follow-up surveillance can track disease progression and provide real-world evidence for future strategy adjustment.

More high-quality clinical and basic researches should be encouraged. In-depth exploration of potential risk factors, pathogenesis and early intervention measures will provide reliable evidence to improve the overall prevention and management of childhood and adolescent DR.

## 5 Conclusion

In summary, elevated HbA1c was associated with DR occurrence and progression in children and adolescents, but this relationship was affected by high heterogeneity and publication bias, becoming non-significant after bias correction. Exposure metric, sample size, and region were the main sources of heterogeneity. The effects of blood pressure and diabetes duration were unstable and need validation in larger studies. Strict glycemic control and stratified screening remain clinically important for pediatric DR prevention.

## Data Availability

The original contributions presented in the study are included in the article/supplementary material, further inquiries can be directed to the corresponding author.
