# Supplementary material for: Risk factors for the occurrence and progression of diabetic retinopathy in children and adolescents: a systematic review and meta-analysis
Source: Front Public Health. 2026 May 21;14:1832691. doi: 10.3389/fpubh.2026.1832691 (PMC13235689; doi:10.3389/fpubh.2026.1832691)
Supplement: Supplementary file 1 [file Data_Sheet_1.docx]

- PubMed Search Strategy

(("diabetic retinopathy"[MeSH Terms] OR "diabetic retinopathy"[Title/Abstract] OR "retinopathy, diabetic"[Title/Abstract]) AND ("child"[MeSH Terms] OR "adolescent"[MeSH Terms] OR "young adult"[MeSH Terms] OR "pediatric"[Title/Abstract] OR "paediatric"[Title/Abstract] OR "childhood"[Title/Abstract] OR "adolescence"[Title/Abstract] OR "youth"[Title/Abstract]) AND ("diabetes mellitus"[MeSH Terms] OR "diabetes"[Title/Abstract] OR "juvenile diabetes"[Title/Abstract] OR "childhood-onset diabetes"[Title/Abstract])) AND ("risk factor"[Title/Abstract] OR "predictor"[Title/Abstract] OR "incidence"[Title/Abstract] OR "progression"[Title/Abstract])

- Web of Science Search Strategy (Topic Field)

1 Topic "diabetic retinopathy" OR "retinopathy, diabetic" OR "proliferative diabetic retinopathy" OR "non-proliferative diabetic retinopathy"

2 Topic "child" OR "children" OR "adolescent" OR "adolescents" OR "youth" OR "pediatric" OR "paediatric" OR "childhood" OR "adolescence" OR "juvenile"

3 Topic "diabetes" OR "diabetes mellitus" OR "type 1 diabetes" OR "type 2 diabetes" OR "juvenile diabetes" OR "childhood-onset diabetes"

4 Topic "risk factor" OR "predictor" OR "incidence" OR "progression" OR "risk" OR "associated factor"

Combination: Lines 1 AND 2 AND 3 AND 4

Search link: <https://www.webofscience.com/wos/woscc/summary/9b87b346-4d86-4520-9611-5fc3f5d1d7e8-01a404daef/relevance/1>

- Embase Search Strategy

#1 'diabetic retinopathy'/exp OR "diabetic retinopathy":ti,ab OR "retinopathy, diabetic":ti,ab OR "proliferative diabetic retinopathy":ti,ab OR "non-proliferative diabetic retinopathy":ti,ab

#2 'child'/exp OR 'adolescent'/exp OR "pediatric":ti,ab OR "paediatric":ti,ab OR "childhood":ti,ab OR "adolescence":ti,ab OR "youth":ti,ab OR "juvenile":ti,ab

#3 'diabetes mellitus'/exp OR "diabetes":ti,ab OR "type 1 diabetes":ti,ab OR "type 2 diabetes":ti,ab OR "juvenile diabetes":ti,ab OR "childhood-onset diabetes":ti,ab

#4 "risk factor":ti,ab OR "predictor":ti,ab OR "incidence":ti,ab OR "progression":ti,ab OR "risk":ti,ab OR "associated factor":ti,ab

#5 #1 AND #2 AND #3 AND #4

- Scopus Search Strategy

ALL("diabetic retinopathy" OR "proliferative diabetic retinopathy") AND ALL("child" OR "pediatric") AND ALL("diabetes" OR "type 1 diabetes") AND ALL("risk factor" OR "progression")

Note: This initial broad search retrieved 5390 records. Further document type restriction was conducted within the Scopus database platform, and only original research articles were retained, resulting in a final number of 1862 records for subsequent screening.
